# A matheuristic for customized multi-level multi-criteria university timetabling

**DOI:** 10.1007/s10479-023-05325-2

**Published:** 2023-04-07

**Authors:** Fabian Dunke, Stefan Nickel

**Affiliations:** Institute for Operations Research, Discrete Optimization and Logistics, Karlsruhe Institute of Technology, Kaiserstr. 12, 76131 Karlsruhe, Germany

**Keywords:** University timetabling, Matheuristic, Student scheduling, Multi-criteria decision making, Artificial neural network meta-model

## Abstract

Course timetables are the organizational foundation of a university’s educational program. While students and lecturers perceive timetable quality individually according to their preferences, there are also collective criteria derived normatively such as balanced workloads or idle time avoidance. A recent challenge and opportunity in curriculum-based timetabling consists of customizing timetables with respect to individual student preferences and with respect to integrating online courses as part of modern course programs or in reaction to flexibility requirements as posed in pandemic situations. Curricula consisting of (large) lectures and (small) tutorials further open the possibility for optimizing not only the lecture and tutorial plan for all students but also the assignments of individual students to tutorial slots. In this paper, we develop a multi-level planning process for university timetabling: On the tactical level, a lecture and tutorial plan is determined for a set of study programs; on the operational level, individual timetables are generated for each student interlacing the lecture plan through a selection of tutorials from the tutorial plan favoring individual preferences. We utilize this mathematical-programming-based planning process as part of a matheuristic which implements a genetic algorithm in order to improve lecture plans, tutorial plans, and individual timetables so as to find an overall university program with well-balanced timetable performance criteria. Since the evaluation of the fitness function amounts to invoking the entire planning process, we additionally provide a proxy in the form of an artificial neural network metamodel. Computational results exhibit the procedure’s capability of generating high quality schedules.

## Introduction

In view of a steadily increasing number of student enrollments in universities over the past decades, the efficient utilization of existing university resources such as rooms, time availabilities, and capacities has become a sine qua non for the successful scheduling of university course programs (Daskalaki et al., 2004; Bettinelli et al., 2015). The importance of an efficient resource utilization is further catalyzed by aging effects and related renovation needs of university facilities (Shiue et al., 2019). In Germany, a large share of the buildings has been erected during the 1960 s to 1980 s. Most recently, the coronavirus pandemic has brought forth additional requirements on course scheduling such as the necessity to integrate on-site and online teaching into the schedule or to change the structure schedule (Barnhart et al., 2022). From a complexity perspective, the discipline of university scheduling hosts numerous difficult combinatorial problems from the class of $\mathcal{N}\mathcal{P}$-hard optimization problems (Bettinelli et al., 2015; Goh et al., 2017) making university scheduling computationally hard.

In practice, university timetabling is mainly deployed on the tactical and operational planning level (Daskalaki & Birbas, 2005; Babaei et al., 2015; Lindahl et al., 2017). Base schedules are generated several weeks ahead of the semester with opportunities for last-minute changes in individual student schedules. Regarding the academic practice, there are notable differences on which problem setting is considered and which solution methodology is to be deployed. This results in a diverse organization of the field and the unavailability of standard solution procedures (Burke et al., 1997; Jat & Yang, 2011; Bettinelli et al., 2015; Teoh et al., 2015). However, it also becomes obvious that several archetypical types of class appointments exist which can be distinguished from each other. This particularly holds for lectures and tutorials where the former appointment is to be visited by all students of a study program, whereas the latter appointment is visited by smaller student groups but offered in larger frequency (Carter & Laporte, 1997; Schaerf, 1999; Müller & Murray, 2010; Bowman, 2021).

**Fig. 1:**
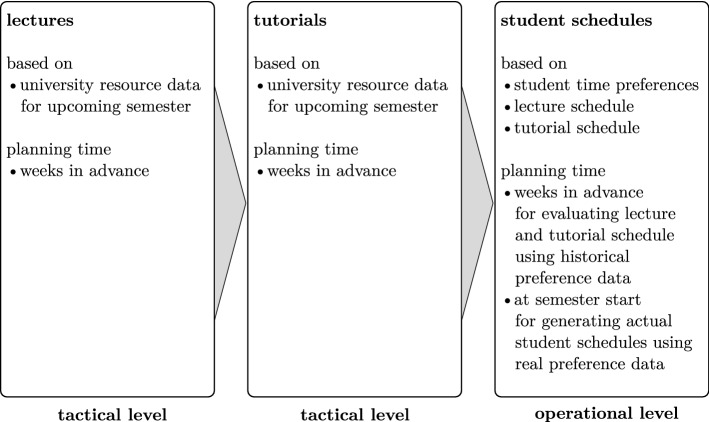
Multi-level university timetabling

In this paper, we extend the classical scope of university scheduling mainly directed at tactical lecture planning to the operational level by generating ad-hoc individual student schedules once they specify their time preferences in the beginning of the semester. To have the possibility of finding promising lecture and tutorial schedules already in the tactical phase—which serves as an input to the operational phase—we simulate student time preferences in the tactical phase to obtain an estimate for the anticipated schedule quality on the individual student level. Figure 1 summarizes the multi-level character of this process. Observe that the third level (operational planning of individual student schedules) is executed at two points in time: First, during the planning phase several weeks ahead of the semester using sampled preference data; second, immediately at the semester start using real student preference data to produce the actual individual schedules for the students.

To tackle the task of multi-level university timetabling methodologically, we employ an advanced matheuristic approach. The overarching solution process is organized by a genetic algorithm (GA) controlling the search for a favorable university schedule. The GA exhibits integrated calls to solving integer programming (IP) formulations of the lecture scheduling, tutorial scheduling, and individual student scheduling problem. Since there are several objectives (e.g., perceived schedule quality, number of gaps in schedules, number of lectures per day) with differing relevance to different stakeholders (students, lecturers, didactic quality control) encountered at different levels of the planning process, there is no straight-forward way of defining a single objective function. Rather, practical experience of timetable planners is necessary to calibrate resulting schedules to student and lecturer needs. Therefore, solution proposals in the form of schedules are evaluated by a multi-dimensional vector of performance criterion values. To extend the search range this outline is enhanced with a metamodel serving as a predictor for schedule performance. The use of such a metamodel (e.g., in the form of an artificial neural network (ANN)) provides a computational cheap way of determining schedule performance on an approximative basis. This functionality is exploited algorithmically in the course of the GA to check solution proposals and include them only when sufficiently good performance can be expected.

The paper contributes a framework for multi-level multi-criteria university timetabling well-suited for contemporary needs of academic personnel and students. Due to its matheuristic character, the framework is adaptable to additional requirements which might emerge under changing circumstances, e.g., in case of sudden pandemic activity. The proposed approach to university scheduling represents a major advancement in terms of exploiting existing optimization potential. Practically, this leads to a heightened level of service quality for students in the form of customized timetables considering individual preferences as well as an efficient utilization of available resources needed to comply with didactic standards and best practices. As shown by the numerical experiments for the case of a faculty-wide scheduling of four study programs with 1950 students, the developed outline yields immense practical benefit to timetable planners reducing manual planning efforts substantially.

The paper remainder is organized as follows: Sect. 2 reviews literature on topics related to multi-level university timetabling. Section 3 develops a metamodel-enhanced matheuristic in the form of a GA for multi-level multi-criteria university timetabling. In Sect. 4, we demonstrate the applicability of the method based on real world and synthesized data. To foster best practices in the field of multi-level university timetabling, Sect. 5 summarizes key findings derived throughout the development and application of the methodology. Finally, Sect. 6 closes with a discussion of remaining challenges.

## Literature review

University timetabling problems arise in many different variants. Nonetheless, over the past two decades the field has received structuring due to regular international timetabling competitions [ITC, cf. Di Gaspero et al. (2007); Bettinelli et al. (2015)]. The first two editions (ITC 2003/2007) also aimed at promoting and organizing the overall field of educational timetabling, whereas the third edition (ITC 2011) paid attention to high school timetabling. It was then the fourth edition (ITC 2019) which specifically focused on a more complex university timetabling problem so as to bring the discipline closer to reality (Müller et al., 2018, 2022). In particular, the 2019 setting combined elements of classical room-time assignments with student sectioning under a given hierarchy of course relations accommodating different class types (such as lectures, recitations, laboratories). Further, it allowed for weekly schedule changes within a fine-grained time resolution of five minute periods. Despite several similarities with this paper (such as the integration of different class types and student sectioning), there are also substantial differences (such as the multi-level approach with several planning stages, base schedules for large study programs, consideration of multiple objectives, methodological treatment as an ANN-enhanced matheuristic, stakeholder participation through consideration of lecturer and student preference data). Nonetheless, the most successful solution methods are in line with those selected in our research. More specifically, the winners connect graph theory and mathematical programming (Holm et al., 2022) to build a matheuristic based on initial solution construction, fix-and-optimize enhancements, and bounding procedures (Mikkelsen & Holm, 2022). Likewise, the second place winners utilize integer programming with reduction procedures (Rappos et al., 2022). Runners-up then employ a MaxSAT solver (Lemos et al., 2022) and a simulated annealing metaheuristic (Sylejmani et al., 2022). Overall, concerning solution techniques, integer programming (IP) and metaheuristics form the largest share, followed by constraint logic programming (CLP), graph coloring, and case-based reasoning. The subsequent literature review accounts for these developments. We recall that a matheuristic is a solution method employing IP models within a heuristic framework. Since this notion is in frequent use only since the 2010 s, many prior works exist without explicitly referencing this notion.

### Overviews on educational and university timetabling

An early introduction and categorization of educational timetabling is given by de Werra (1985). Differences between problem types and their relation to each other as well as to solution approaches like IP and graph theory are figured out and anticipate the field’s upcoming evolution resulting from improving computational capabilities. Carter and Laporte (1997) organize the field into course timetabling, class-teacher assignment, student scheduling, teacher assignment, and classroom assignment. Concerning course timetabling, they distinguish between master scheduling and demand-driven scheduling. The former is coined by a prior determination of all course schedules, whereas the latter is driven by initial student choices of courses and sections. Motivated by study program modularity and competing stakeholder interests (administrators, students, departments), Burke et al. (1997) identify the need for standardization. They classify constraints both with respect to their meaning (capacities, assignments, timing, coherence) and degree of allowed violation (hard, soft). Concerning solution methods, they identify metaheuristic methods and CLP as most promising for automation. Schaerf (1999) contribute an extensive survey on automated timetabling for university, school, and examination timetabling. Each class is discussed in terms of complexity, formulations, extensions, and solution methods. Since all problems are computationally difficult, different solution techniques (IP, graph theory, tailored (meta-) heuristics) are considered. Standardization, devoted analysis of specific solution methods, and combination of solution methods are suggested for further research. Burke and Petrovic (2002) focus on solution methods distinguishing between sequential, cluster, constraint-based, and metaheuristic methods. An in-depth analysis is carried out for combined heuristics and meta-heuristics which had proven successful in practice. Multi-criteria optimization methods are suggested to account for hard and soft constraints systematically. The authors also indicate the need for standardized automated approaches to propel further development. The ultimate goal then consists in a generalized framework for the automated selection of algorithms depending on problem characteristics, i.e., a framework tailored for timetabling derived from case-based selection heuristics and hyper-heuristics. McCollum et al. (2010) then introduce for the timetabling competition ITC 2007 the division into curriculum-based course timetabling (CBCT), post-enrollment course timetabling (PECT), and examination timetabling. For the paper at hand, the former two are of interest. In CBCT, schedules are determined based on student curricula without prior consultation of students, whereas in PECT, students upon enrollment first issue their demands for courses and course sections. The ITC was further held in 2002, 2011, and 2019 and contributed definitions and distinctions between problem variants. Information on the 2019 edition focusing on complex university scheduling are found in Müller et al. (2018). MirHassani and Habibi (2013) continue the work from Burke and Petrovic (2002) and re-enter IP as a viable solution methodology due to significant progress in computational capabilities. They conclude that GAs and memetic algorithms on the one hand, and IP on the other hand form the two current main branches of solution methodologies. Babaei et al. (2015) differentiate between operations research methods (IP, graph coloring, constraint satisfaction) and metaheuristics (including GA, tabu search (TS), (variable) neighborhood search, ant colony optimization) and emphasize the necessity for advanced methods such as method combinations or (distributed) multi-agent approaches where the allocation of room and time slots is managed through communication, collaboration and negotiation between departments to constructively deal with the complexity of timetabling.

### IP methods for course timetabling

An early IP approach to course scheduling is conducted by Ferland and Roy (1985) who decompose the problem into two sequential phases (class period scheduling, classroom assignment). Due to their identical mathematical structure, both problems are treated as quadratic assignment problems with penalties for conflicting scheduling decisions. The paper provides a first hint at how IP can be utilized as part of decomposition schemes. Motivated by increased computational power, Daskalaki et al. (2004) present an IP model with general and specific features occurring in the authors’ home institution. Solution retrieval is required to be effective (i.e., complying with the institution’s timetabling rules) as well as satisfactory (i.e., favoring timetables of high perceived quality). To ensure acceptable computing times regardless of problem size, Daskalaki and Birbas (2005) introduce an IP-based two-phase relaxation procedure where in the first step constraints on consecutiveness between lectures are omitted, but re-introduced in the second stage. Hence, only local optima with respect to this second stage can be identified rendering the method a matheuristic. Schimmelpfeng and Helber (2007) devise an IP model to realize a centralized planning approach for a medium-sized business school. Contrasting most other publications, schedule quality is evaluated upon implementation in practice and found to be superior to prior schedules. An IP-based decomposition scheme for CBCT is devised by Lach and Lübbecke (2012). In the first stage, lectures and time slots are matched, whereas in the second stage rooms are assigned. Since the first stage takes implicit care of successive room assignments, the approach is exact and leads to computing time reductions compared to a simultaneous method. Burke et al. (2010) develop a two-stage solution procedure with a coupling between IP and an associated control strategy. In the first stage, a relaxation disregarding room-assignment issues is solved. Violations are then fixed in the second stage to produce locally optimal solutions. Objectives comprise room suitabilities, event spread over the day, desirability of timetabling patterns, and distance between rooms. Van den Broek and Hurkens (2012) develop a matheuristic for PECT based on solving the linear programming relaxation of an IP model through column generation. To correct for infeasibility, the procedure is enhanced by a fixing heuristic. In addition, an improvement heuristic based on an IP formulation accounting for soft constraints is employed to obtain practically competitive schedules. Méndez-Díaz et al. (2016) consider a generalized PECT version. They first formulate an IP model, but since the model is huge in terms of variables and constraints, a two-stage relax-and-fix heuristic building upon this model, i.e., a matheuristic, is proposed. Hoshino and Fabris (2020) present a timetabling problem variant suited to creating timetables satisfying both lecturers’ and students’ quality requirements. They devise a two-stage procedure employing graph coloring in the first stage as a pre-processing to bundle one-section courses, to be followed by solving an IP formulation whose running time is reduced drastically while solution quality only deteriorates marginally using the first stage input.

### Metaheuristic methods for course timetabling

A review classifying university timetabling metaheuristics according to their structure is given by Lewis (2008). One-stage algorithms consider hard and soft constraints simultaneously, two-stage algorithms deal with hard constraints in the first stage before opting for soft constraints in the second stage. Moreover, relaxation algorithms first permit violations of hard constraints which are repaired successively. Another review on metaheuristic approaches to academic scheduling is provided by Teoh et al. (2015). It organizes different classes of metaheuristics (TS, GA, simulated annealing (SA), particle swarm optimization, fuzzy logic algorithms, ant colony optimization) and hyper-heuristics. The different methods are deemed capable of delivering promising solutions; however, sensitivities to method-specific parameter settings differ significantly. To advance timetabling towards automation, Pillay (2016) surveys the discipline of hyper-heuristics where a heuristic is to be devised to choose a suitable heuristic for a problem. Both selection and generation hyper-heuristics are organized into constructive and perturbation (i.e., improvement) hyper-heuristics. Selection algorithms make use of existing heuristics, whereas generation algorithms automatically create new procedures. Pongcharoen et al. (2008) present a framework for metaheuristic university course scheduling involving stochastic elements as encountered in GAs, SA, and random search. The authors recognize the need for including multiple objectives at least via soft constraints to account for interests of several stakeholders. In the presented metaheuristic framework, hard constraints are explicitly ensured through a repair scheme, while multiple objectives are addressed by including them in the objective functions (e.g., fitness evaluation) used by the method.

There is a vast variety of metaheuristics not only in their pure forms, but increasingly also in hybrid approaches. SA is involved in a large share of them. Ceschia et al. (2012) comprehensively explore SA for PECT. They devise an outline which is embedded into several performance-enhancing measures such as preprocessing and constraint reformulation. It makes use of a composite neighborhood structure allowing for event swaps and re-schedulings. Experimentation shows that parameterization can be tuned so as to obtain excelling performance on any instance. Lewis (2012) addresses PECT in a three-stage outline through a rule-based construction process in the first step disregarding one class of hard constraints to be followed by two SA improvement phases with the first one integrating missing hard constraints and the second one considering remaining soft constraints. Goh et al. (2017) combine local search (LS), TS, and SA. Through sampling and perturbation, they develop enhanced TS versions to generate feasible solutions in the first stage. These are then improved in terms of reducing soft constraint violations by SA with reheating and alternative neighborhood exploration. The outline is refined in (Goh et al., 2019) by reinforcement learning aiming at finding a balanced composition of neighborhood structures. Extensive numerical experiments are conducted in Goh et al. (2020). Search strategies form another focus in metaheuristic university timetabling. For a real-world university timetabling problem, De Causmaecker et al. (2009) establish a multi-stage solution procedure where in each stage an additional constraint is fulfilled. The procedure builds upon a LS scheme which employs TS and operates under varying neighborhood types. Suyanto (2010) considers university course timetabling in a two-stage process employing a GA at both stages. In the first stage, student sectioning is omitted such that only the generation of the timetable is considered, whereas in the second stage including student sectioning allows to reduce course conflict violations encountered by individual students. For CBCT, Lü and Hao (2010) introduce an adaptive TS algorithm consisting of initialization, diversification and intensification phases whereof the latter two are combined adaptively to reduce soft constraint violations. The analysis is generalized in Lü et al. (2011) by examining different neighborhood structures based on LS. Criteria according to which neighborhood suitability is assessed comprise percentage of improving neighbors, improvement strength, and search steps. The evaluation of neighborhood capabilities is carried out when these neighborhoods are employed as part of a steepest descent, TS, variable neighborhood search, and iterated LS. Jat and Yang (2011) develop a hybrid metaheuristic approach combining GA with TS. Hybridization results in two algorithmic phases: A guided search GA first delivers a PECT solution which is further improved by TS. The GA is guided through a data structure which memorizes promising solution characteristics encountered in feasible solutions. Cambazard et al. (2012) consider both LS and CLP to find feasible PECT schedules. They first cast the problem as list coloring in an event conflict graph from which an event-to-room matching subproblem is derived for every time slot. The LS then builds upon a coloring strategy. Likewise, alternate colorings and matchings are utilized in the CLP approach. Integrating LS and CLP in an overarching large neighborhood search is most promising in terms of computational requirements. An extensive analysis of different scatter search variants applied to university course timetabling is given by Jaradat et al. (2014). Approaches differ in search strategies and subroutines. Finally, we mention ant colony optimization for PECT by Nothegger et al. (2012). The pheromone information is composed of probabilities for assigning events to time slots and probabilities for assigning events to rooms. The algorithm is enhanced through LS and pheromone amplification.

### Multi-level university timetabling

In contrast to the multi-stage methods discussed above, multi-level university timetabling refers to different organizational levels involved in educational timetabling. The interrelated problems of master timetabling and student section assignment (grouping) are discussed in Aubin and Ferland (1989). They derive an iterative heuristic oscillating between master timetable modification and student section assignment until no further improvement is possible. In doing so, they first define an IP model and then approach it through an iterative heuristic for the timetabling and assignment subproblems. A two-level setting consisting of master timetabling and student sectioning is tackled by Banks et al. (1998) through constraint satisfaction where the problem is modeled heuristically (i.e., not considering all constraints), but solving arising auxiliary problems exactly. The proposed method follows an iterative constraint-adding approach until a master timetable containing multiple course sections is constructed; this is followed by a greedy section assignment to obtain individual student schedules. The complexity of the overall timetabling process is illustrated by Carter (2000) in a comprehensive demand-driven course timetabling and student scheduling system. The setting is decomposed into several subproblems comprising assignment of course sections to times, classroom assignment, and automated student scheduling via section assignments. It is explained how administrative personnel can engage in the system interactively to resolve conflicts and contribute to high quality student schedules. Rudová and Murray (2002) and Rudová et al. (2011) present a practical outline for complex university timetabling at Purdue University. The setting is coined by many institution-specific requirements illustrating the political dimension of timetabling. The approach encompasses all practically necessary steps such as establishing the course structure, translating it into classes upon which constraints are imposed, and application of algorithms from constraint satisfaction and CLP allowing for partial satisfaction of soft constraints. Further, the method hosts a structure to incorporate changes interactively. The authors conclude that the method contains several elements amenable to generalization. A holistic approach to student sectioning is taken by Müller and Murray (2010) who consider three different problem variants, namely in parallel to establishing the master course timetable (initial sectioning), after establishing the master course timetable (batch sectioning), and as an online method to correct conflicts or incorporate changes into student schedules (online sectioning). These problems can be addressed successively as part of the planning cycle. Devised algorithms build upon constraint satisfaction and optimization with the online algorithm incorporating rule-based decision making. A comprehensive view on problems related to timetabling on the strategic, tactical, and operational level is given by Lindahl et al. (2017) who provide a broadened perspective including course assignment, room planning, teaching periods, and quality recovering. In particular, input–output relations between different problems and hence the interplay of decisions on an organizational level are derived. Developed methods are based on IP leading to fix-and-optimize matheuristics and a Pareto efficiency approach to quality recovering under disruptions induced by operational changes. To analyze the availability of different resources and thereby to assess the impact of strategic decisions onto the operational level, an $\epsilon$-method algorithm for bi-objective optimization is invoked.

### Multi-objective university timetabling

Apart from addressing multiple objectives by minimization of a single substitute objective or in a two-stage approach with soft constraints handled in the second stage as discussed previously (Burke et al., 2010; Lewis, 2012; Goh et al., 2017, 2019, 2020), research focusing on multi-criteria concepts is scarce which could be due to the large number of interests and stakeholders involved in educational institutions. Burke et al. (2005) give an overview on multi-objective metaheuristics for scheduling and timetabling. For educational timetabling, they find that multi-objective metaheuristics, in particular evolutionary approaches, are used scarcely. Carrasco and Pato (2000) first employ an evolutionary multi-objective algorithm, namely a version of the non-dominated sorting GA (NSGA) for educational timetabling with two objectives concerning class- and teacher-oriented violations caused by a schedule. Using tailored definitions of evolutionary operators, they conclude that NSGA successfully yields Pareto schedules outperforming manual schedules. As an advancement, Datta et al. (2007) invoke NSGA-II (which improves NSGA by fast non-dominated sorting, increased elitism, and parameterless diversification) for university timetabling with the two objectives of minimizing the number of free intermediate time slots for students and consecutive lectures for lecturers. The algorithm yields high quality schedules for a real world case in India such that the authors recommend its use due to the aptness for complex settings. The authors further augment the evolutionary outline of forming problem-specific versions of NSGA-II to the general field of resource allocation problems (Datta et al., 2008). Jat et al. (2011) enhance NSGA-II through hybridization in the form of two LS procedures which aim at exploring the trade-off space through a guided search for the offspring generation. This is accomplished by storing solution components of previously found promising schedules to be re-utilized. Another hybridized variant of NSGA-II is established for PECT by Lohpetch and Jaengchuea (2016). Hybridization is achieved by integrating LS and TS into the overarching NSGA-II. Soft constraints address the number of violations of requirements on the minimum number of events, consecutive events, and end-of-day events of students. Likewise, in Abdullah et al. (2010), a multi-objective version of PECT is tackled through a specifically tailored NSGA-II variant. The objective considered affects the minimization of the soft constraints including the avoidance of gaps in individual student schedules and the avoidance of days with only a single course. Akkan and Gülcü (2018) and Akkan et al. (2022) consider robustness as a second dimension besides schedule quality. Robustness measures—also inspired by the general idea of slack—are defined to account for input data changes. The resulting bi-criteria problem is tackled by a GA combined with hill climbing and SA. A different solution concept is formed by multi-objective hyper-heuristics (Silva et al., 2004). The outline intends to compose a heuristic by selecting in each step a neighborhood search heuristic and organizing the procedure in an overarching TS so as to exclude neighborhoods temporarily. The approach shows promising results for university course timetabling.

## Methodology

**Fig. 2:**
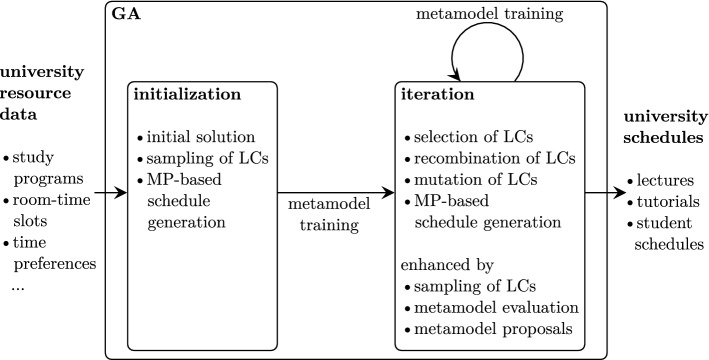
Matheuristic in the form of a metamodel-enhanced GA

Multi-level multi-criteria university timetabling aims at determining weekly individual schedules for all students of considered study programs. We develop a matheuristic by combining IP, metaheuristics, and metamodels in a tailored solution outline accounting both for multiple planning levels (lecture schedules, tutorial schedules, student schedules) as well as for multiple objectives (lecture schedule quality perceived by lecturers/students, ideal number of lectures per day and study program, avoidance of gaps between scheduled courses, avoidance of days with one or none class). Specifically, we use IP models for determining concrete lecture schedules, tutorial schedules, and student schedules. However, since there are several objectives relevant to several stakeholders (lecturers, students, study program instructors), the different objectives yielded by solving the IP formulations must be taken into account collectively. This is achieved within a GA outline which repeatedly generates populations of lecture configurations to be optimized into university schedules. To this end, we define a lecture configuration (LC) as a specification of the number of lectures per study program and week day (cf. also Sect. 3.1). Hence, the GA serves as a means to ensure diversification of schedules with regards to the objectives. The input–output data collected in this process occurs in the form of pairs consisting of a lecture configuration and corresponding objective values. This data can be used to train a metamodel—for instance an ANN—allowing to directly provide estimates of prospective schedule objective values without having to determine all schedules through solving the IP models. This facilitates a computationally attractive pre-selection of lecture configurations from which only promising candidates are further considered in larger detail. This solution outline is summarized in Fig. 2.

### Nomenclature and terminology

We first present the nomenclature to ensure a common understanding of all used terms and entities. This is also necessary since the setting differs from classical university timetabling through the multi-level aspect. The presentation requires data on the following three aspects: study programs, resources (rooms, time availabilities), student preferences.

- **study program, curriculum, student** A study program consists of a set of courses, called curriculum, and has a specific number of enrolled students. A student is enrolled in a specific study program and has to visit all of its courses.
- **course, lecture, tutorial, class** A course consists of at least one lecture and can have several tutorials. Lectures are for large student groups, tutorials are for smaller student groups such that there must be several tutorials for a course with tutorials. Class is the collective term for lectures and tutorials. A lecture can be held either on-site (i.e., in personal attendance) or online; for tutorials we assume only on-site classes. Every class takes place weekly.
- **time slot, room-time slot, day** A time slot corresponds to a specific two-hour period and can be on each day from 08–10, 10–12, 12–14, 14–16, 16–18, or 18–20 o’clock. A room-time slot is a pair of a specific room and a specific time slot. A room has a maximum capacity. Online lectures take place in a virtual room with infinite capacity. A day is Mon, Tue, Wed, Thu, Fri, i.e., only week days are considered.
- **appointment, schedule** An appointment is the booking of a class to a room-time slot. A schedule is a set of appointments. Since every class takes place weekly, schedules are repeated weekly.
- **score** A score is an assessment of a time slot with a value from $\{1, 2, 3, 4, 5\}$ and can be given by a student or a lecturer. Higher scores are considered better than lower scores.
- **(valued) lecture configuration** A lecture configuration (LC) gives for each day and study program the number of lectures to be attended by the students of the study program on this day. Hence, a lecture configuration defines the per-day lecture workload of a study program on a coarse level without mentioning which lectures take place at exactly which time slots. A valued lecture configuration (VLC) is a pair consisting of an LC and its associated objective value vector for the multiple schedule criteria.

The notion of a room-time slot is introduced for two reasons: First, it allows to specify explicitly when a room is available which is relevant whenever a room exhibits limited availability as is typical for large lecture halls. Second, it simplifies notation in terms of storing information about which class takes place at which time in which room. This is useful when formulating models in later stages of the planning process using data input from earlier stages. We remark that for tutorials we only consider on-site tutorials as—due to the enormous number of tutorial appointments—it is neither possible nor reasonable to ensure commuting reductions for small fractions of each study program’s students as these student fractions are composed differently for each tutorial appointment.

### IP models for lecture, tutorial, and student schedule planning

We first explain the sequential approach to the generation of lecture schedules, tutorial schedules, and individual student schedules via IP. The outline generates feasible schedules which address the different schedule performance criteria. Due to the lack of a crisp solution concept for the multi-criteria setting in the face of a large number of heterogeneous performance criteria, we utilize the set of generated schedules as part of the population in the GA later on described in Sect. 3.3. The sequential approach of solving the IP models not only results from the practical procedure of university timetabling, but also from several other reasons: First, lecture appointments apply to all students and they are subject to the tightest resource limitation in terms of available lecture halls. Secondly, there is a different granularity in terms of modeling elements in the different models; together with different objective functions coming into effect at different stages of planning this makes a combined perspective both prohibitive and erroneous. Finally, computational efforts can be reduced substantially. Therefore, we next present the mathematical models for the three planning steps: The lecture schedules of study programs represent the skeleton of the university schedule and are determined first. Tutorial plans are then generated in accordance with the previously determined lecture schedules. Finally, individual student schedules assign each student individual tutorial appointments.

#### Lecture schedule planning

**Fig. 3:**
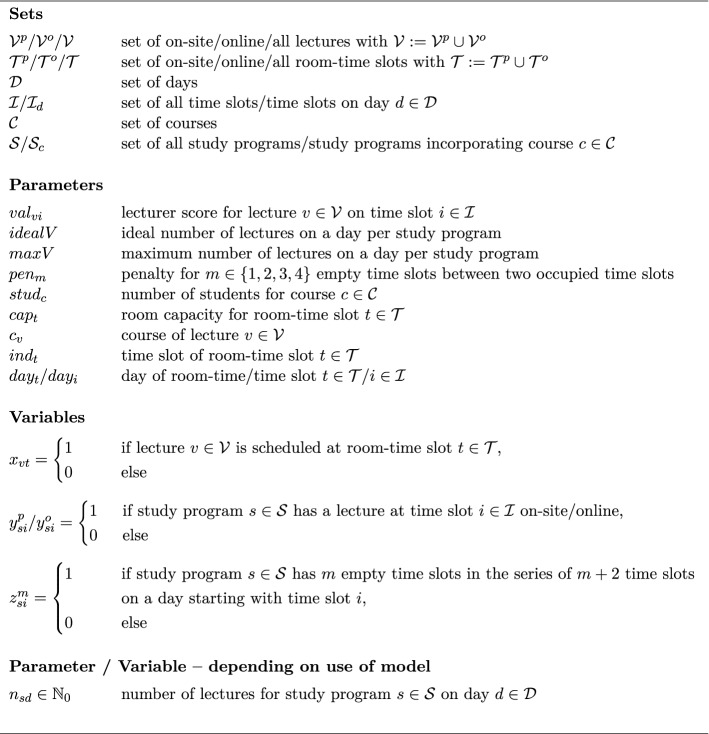
Notation for the IP formulation of the lecture planning problem

**Fig. 4:**
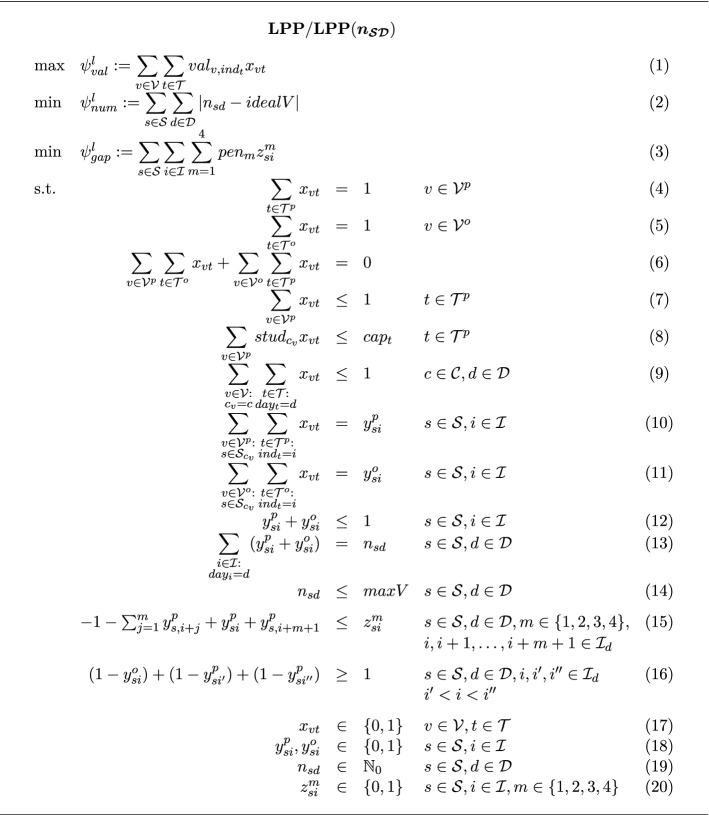
IP formulation of the lecture planning problem

In the first step, lecture schedules are generated for all study programs such that there are no lecture conflicts for any student. The notation for the lecture schedule planning problem $\textbf{LPP}$ is summarized in Fig. 3. Depending on whether the LCs $n_{\mathcal{S}\mathcal{D}}=(n_{sd})_{s \in \mathcal S, d \in \mathcal D}$ are passed as parameters to the IP model in Fig. 4, either model $\textbf{LPP}$ with $n_{\mathcal{S}\mathcal{D}}=(n_{sd})_{s \in \mathcal S, d \in \mathcal D}$ as decision variables or model $\textbf{LPP}(\varvec{n_{\mathcal{S}\mathcal{D}}})$ with $n_{\mathcal{S}\mathcal{D}}=(n_{sd})_{s \in \mathcal S, d \in \mathcal D}$ as parameters is solved.

Three objectives are considered as performance measures for lecture schedules. First, $\psi ^{l}_{val}$ gives the sum of the realized lecturer scores indicating the lecturers’ satisfaction with the generated schedule. The second objective $\psi ^{l}_{num}$ serves as a normative didactic measure in order to track that there are no workload overloads in the study programs’ curricula. Thirdly, $\psi ^{l}_{gap}$ defines a performance measure penalizing empty gaps (i.e., time slots without lectures) in the lecture schedules since such gaps unavoidably imply discontentment for all those students for which such a hole cannot be filled with a tutorial appointment. Hence, the third objective indirectly measures the students’ satisfaction with the generated schedule. We use a weighted sum of these three objectives as the objective function for solving the IP model. The weight factors are determined through numerical experimentation such that promising objective values are attained for all objectives. There is no further meaning of the weighting due to the heterogeneity of the objectives involved.

Objective (1) gives the total lecturer score. Objective (2) measures the total deviation from the ideal number of lectures per day. Observe that the absolute value function in objective (2) is automatically converted to a linear expression in contemporary IP solvers. Objective (3) yields the total penalty for empty time slots of specific lengths between two consecutive lectures. Constraints (4), (5), and (6) ensure that each on-site and online lecture receives exactly one on-site or online room-time slot, respectively. For each on-site room-time slot, constraint (7) only allows at most one lecture to be assigned to it. Constraint (8) delimits the capacity utilized by the students which have to attend the on-site lectures to the room’s seating capacity. Constraint (9) prescribes that for each course at most one of its lectures takes place on a single day. Constraints (10) and (11) define the class indicators, i.e., the occurrence of an on-site lecture or online lecture for a specific study program on a specific time slot, respectively. The class indicators are used in constraint (12) to prohibit simultaneous on-site and online lectures for a study program. Constraint (13) defines lecture counts, i.e., the number of lectures of a study program on a specific day. It is forbidden to exceed a maximum allowable number of lectures per day through constraint (14). Constraint (15) (explained in more detail below) forces the indicating variable for the series of empty slots to 1 whenever a series of empty slots occurs. Together with objective (3) the variable is set to 0 when such a series of empty slots does not occur. Constraint (16) (explained in more detail below) ensures that students do not have to go twice to the campus on a day by prohibiting that an online lecture takes place between two on-site lectures on a day.

We explain constraints (15) and (16) in detail as their validity is not seen obviously. Constraint (15) has to ensure that for each study program a block of *m* empty time slots in between two on-site lectures is recognized and indicated in the *z*-variable such that it can be penalized by the objective function. For simpler notation, we omit the index of the study program and the suffix indicating on-site lectures. Since $z_i^m$ is minimized in the objective function, the constraint only has to ensure that in case of $y_i^p=y_{i+m+1}^p=1$, $y_{i+1}^p=y_{i+m}^p=0$ it follows that $z_i^m = 1$, i.e., in this situation $z_i^m=1$ is enforced whereas in any other situation $z_i^m \in \{0, 1\}$ such that the objective will avoid $z_i^m$=1. With $y=1 \Leftrightarrow \textbf{y}$ and $y=0 \Leftrightarrow \lnot \textbf{y}$, we transform this implication to disjunctive normal form using the material conditional ($\textbf{y} \Rightarrow \textbf{z} \,\,\Leftrightarrow \,\, \lnot \textbf{y} \vee \textbf{z}$) and De Morgan’s second rule ($\lnot (\textbf{y} \wedge \textbf{z}) \,\,\Leftrightarrow \,\, \lnot \textbf{y} \vee \lnot \textbf{z}$):

$$\begin{aligned}&\textbf{y}_i \wedge \textbf{y}_{i+m+1} \wedge \lnot \textbf{y}_{i+1} \wedge \cdots \wedge \lnot \textbf{y}_{i+m} \Rightarrow z_i^m\\ \Leftrightarrow \quad&\lnot (\textbf{y}_i \wedge \textbf{y}_{i+m+1} \wedge \lnot \textbf{y}_{i+1} \wedge \cdots \wedge \lnot \textbf{y}_{i+m}) \vee \textbf{z}_i^m\\ \Leftrightarrow \quad&\lnot \textbf{y}_i \vee \lnot \textbf{y}_{i+m+1} \vee \textbf{y}_{i+1} \vee \cdots \vee \textbf{y}_{i+m} \vee \textbf{z}_i^m\\ \Leftrightarrow \quad&(1-y_i) + (1-y_{i+m+1}) + y_{i+1} + \ldots + y_{i+m} + z_i^m \ge 1\\ \Leftrightarrow \quad&-1 + y_i + y_{i+m+1} - \displaystyle \sum _{j=1}^m y_{i+j} \le z_i^m \end{aligned}$$

Constraint (16) schedules an online lecture such that for each study program containing this lecture it is the first or last lecture of the day. This constraint is due to the requirement that students should not have to go twice to the university campus on a day. For simpler notation, we omit the index of the study program. Hence, the situation which is infeasible on day $d \in \mathcal D$ can be described as having an online lecture on time slot $i \in \mathcal I_d$, an on-site lecture at some earlier time slot $i' \in \mathcal I_d$ of the same day, and another on-site lecture at some later time slot $i'' \in \mathcal I_d$ of the same day. Thus, for $i'< i < i''$, we have to ensure that $y^o_i = 1$ does not come with $y^p_{i'}=1$ and $y^p_{i''}=1$. With $y=1 \Leftrightarrow \textbf{y}$ and $y=0 \Leftrightarrow \lnot \textbf{y}$, we transform this implication to disjunctive normal form using the material conditional ($\textbf{y} \Rightarrow \textbf{z} \,\,\Leftrightarrow \,\, \lnot \textbf{y} \vee \textbf{z}$) and De Morgan’s second rule ($\lnot (\textbf{y} \wedge \textbf{z}) \,\,\Leftrightarrow \,\, \lnot \textbf{y} \vee \lnot \textbf{z}$):

$$\begin{aligned}&\textbf{y}^o_i \Rightarrow \lnot (\textbf{y}^p_{i'} \wedge \textbf{y}^p_{i''})\\ \Leftrightarrow \quad&\textbf{y}^o_i \Rightarrow \lnot \textbf{y}^p_{i'} \vee \lnot \textbf{y}^p_{i''}\\ \Leftrightarrow \quad&\lnot \textbf{y}^o_i \vee (\lnot \textbf{y}^p_{i'} \vee \lnot \textbf{y}^p_{i''})\\ \Leftrightarrow \quad&\lnot \textbf{y}^o_i \vee \lnot \textbf{y}^p_{i'} \vee \lnot \textbf{y}^p_{i''}\\ \Leftrightarrow \quad&(1-y^o_i) + (1-y^p_{i'}) + (1-y^p_{i''}) \ge 1 \end{aligned}$$

We preserve the values of the lecture occurrence indicators to indicate that all students of a study program are occupied when a lecture is scheduled for this study program. To this end, we denote with $s_{p'} \in \mathcal S$ the study program attended by student $p' \in \mathcal P$ with $\mathcal P$ being the set of all students, and we set the student occupation indicator as

$$\begin{aligned} occ_{p'i}&= {\left\{ \begin{array}{ll} 1 &{} \text {if } y_{s_{p'}i}^p = 1 \vee y_{s_{p'}i}^o = 1 \text { for }p' \in \mathcal P, i \in \mathcal I\\ 0 &{} \text {else.} \end{array}\right. } \end{aligned}$$

In $\textbf{LPP} / \textbf{LPP}(\varvec{n_{\mathcal{S}\mathcal{D}}})$, it is tacitly assumed that a lecturer is allocated to exactly one course such that as a result of constraint (9), it is impossible that there will be a time slot clash for any lecturer. This is due to the practical assumption that lecture schedules are required for large study programs in the first place. Therefore, there is agreement that a lecturer should not be assigned to more than one course with such large participant numbers; contrarily, the workload shall be fairly distributed among the teaching personnel. Nonetheless, the model can be easily adapted to permit lecturers being assigned two or more courses. To this end, let $\mathcal L$ be the set of all lecturers and let $lec_v \in \mathcal L$ be the lecturer assigned to lecture $v \in \mathcal V$. Then the constraint

$$ \displaystyle \sum _{\begin{array}{c} v \in \mathcal V:\\ lec_v = l \end{array}}\displaystyle \sum _{\begin{array}{c} t \in \mathcal T:\\ ind_t = i \end{array}} x_{vt} \le 1 \qquad l \in \mathcal L, i \in \mathcal I $$

ensures that a lecturer is not prescribed more than one lecture appointment in each time slot, i.e., time slot clashes for lecturers are ruled out.

#### Tutorial schedule planning

**Fig. 5:**
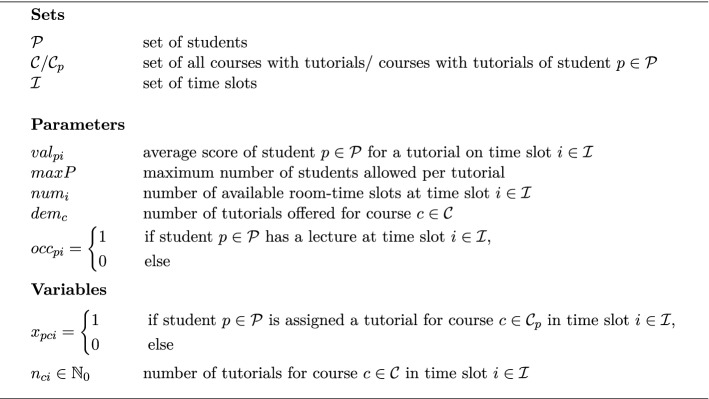
Notation for the IP formulation of the tutorial planning problem

Based on the student occupation indicators $(occ_{pi})_{p \in \mathcal P, i \in \mathcal I}$, tutorials for each course are distributed to the available room-time slots in the step of tutorial schedule planning. The notation for the tutorial schedule planning problem $\textbf{TPP}$ is summarized in Fig. 5.

Tutorials are conducted by student tutors from different semesters and different study programs. Therefore, it can neither be anticipated nor estimated what the tutor time preferences will be. The same holds true for the individual student assessments of the different time slots. These will also be revealed at semester weeks later. As a result, only the average overall student satisfaction is used as a performance criterion in objective $\psi ^{t}_{val}$. It utilizes the historical average of student evaluations for tutorial appointments at the respective time slots of the week. To this end, parameter $val_{pi}$ holds the historical average score of a student $p \in \mathcal P$ enrolled in study program $s_p \in \mathcal S$ for time slot $i \in \mathcal I$.

**Fig. 6:**
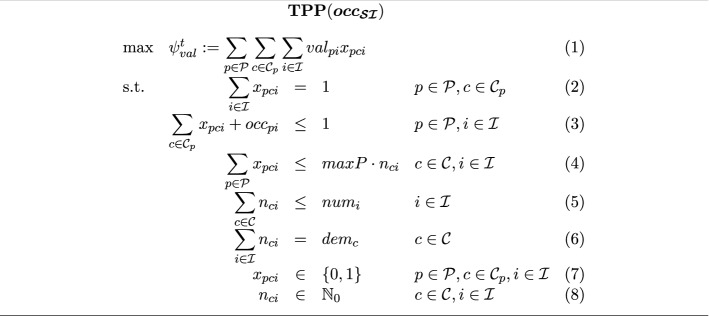
IP formulation of the tutorial planning problem

Even though tutorial appointment participants will be assigned operationally at semester start, the planning process for tutorial appointments which is carried out tactically several weeks before semester start must incorporate individual student enrollments to ensure feasibility of the established tutorial appointments. As a result, we do not only establish a packing of students into tutorials, but also respect the temporal aspect to ensure that no student receives two appointments in a time slot. The IP model in Fig. 6 considers in the objective (1) the historical average student score for the respective time slots and gives the schedule’s total score of the students. Constraint (2) ensures that each student is assigned a tutorial time slot for each course with tutorials. Constraint (3) states that a student can have at most one appointment (either lecture or tutorial) in every time slot. Constraint (4) delimits the number of participants in each tutorial appointment as required by the tutorial character of smaller groups. Constraint (5) asserts that the number of tutorials in a specific time slot does not exceed the number of available rooms at this time slot. Constraint (6) prescribes that the demanded number of tutorials of each course is realized.

We assign tutorial room-time slots $\bar{\mathcal T}$ in accordance with the optimal solution of $\textbf{TPP}(\varvec{occ_{\mathcal{S}\mathcal{I}}})$ as shown by the pseudo-code in Algorithm 1:
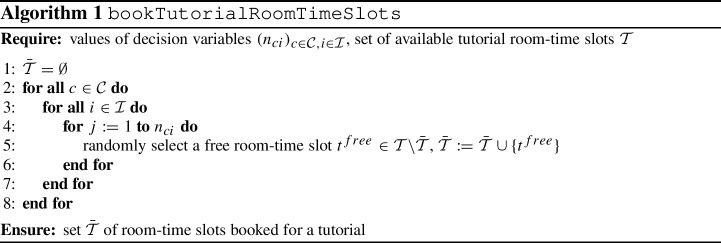


The teaching personnel for the tutorials is typically composed of senior students who are hired exclusively for the tutorials. In particular, these teaching assistants are not known at the tactical planning stage, and they can only be assigned to the available room-time slots operationally at the start of the semester. This can be accomplished, for instance, by solving an assignment problem based on individual scores specified by each student assistant for each booked tutorial room-time slot.

#### Student schedule planning

**Fig. 7:**
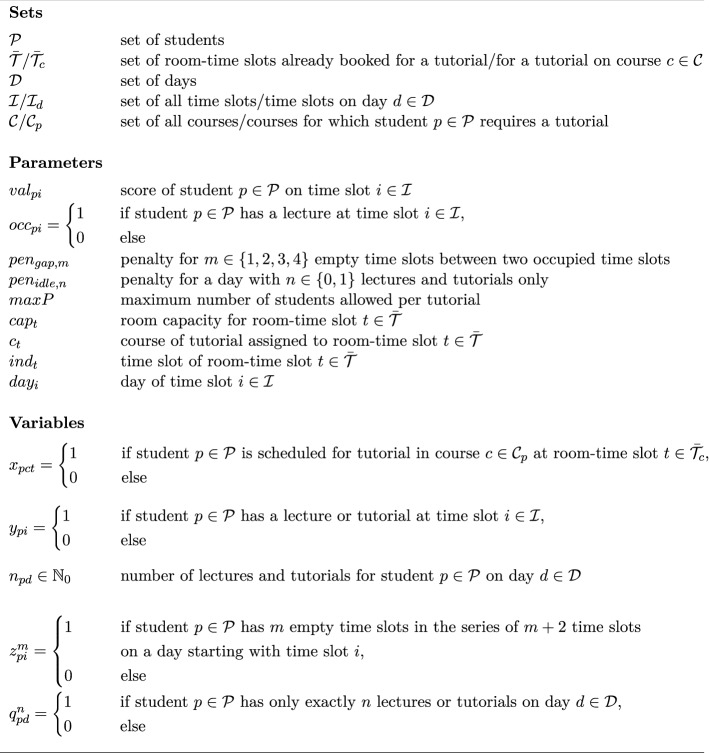
Notation for the IP formulation of the student scheduling problem

**Fig. 8:**
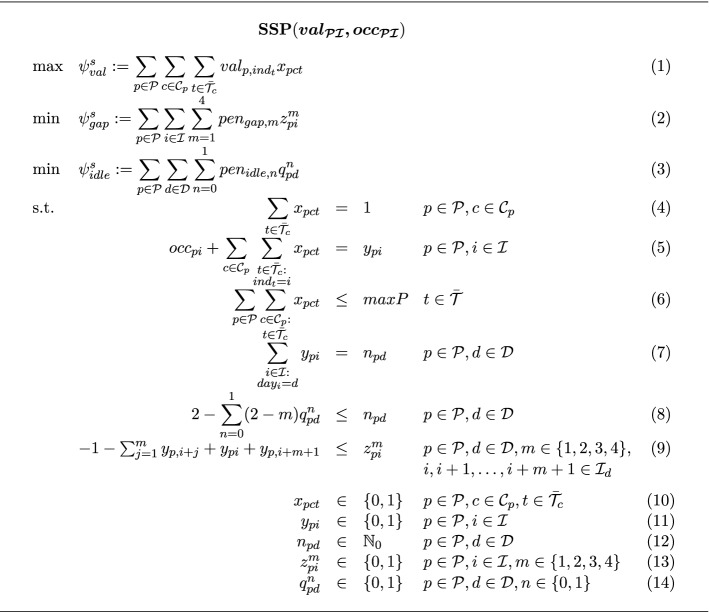
IP formulation of the student scheduling problem

Finally, individual schedules are generated for all students of all study programs simultaneously. For planning purposes, this step is carried out several weeks in advance with simulated student time preferences to check whether the previously determined lecture and tutorial schedules also will allow for satisfactory performance at semester start. In practice, this step is additionally carried out for determining actual student schedules at semester start once students have specified their time slot preferences. The notation for the student schedule planning problem $\textbf{SSP}$ is summarized in Fig. 7. The IP model in Fig. 8 then determines all individual student schedules according to specified time preferences.

The entirety of all student schedules is evaluated by three objectives. First, $\psi ^{s}_{val}$ gives the total realized satisfaction of all students according to their time slot preferences. Secondly, $\psi ^{s}_{gap}$ measures the total penalty incurred by empty slots. Hence, under the assumption that empty slots are undesirable this objective serves as an indirect measure for the students’ satisfaction. Finally, $\psi ^{s}_{idle}$ yields the penalty for days in student schedules which exhibit one or none classes. Hence, this objective serves as a normative didactic measure aiming at a well-balanced distribution of classes over each day. Underutilized days are eliminated such that no student can generate class-free days at the expense of the balance of other students’ schedules. We use a weighted sum of these three objectives as the objective function for solving the IP model. The weight factors are determined through numerical experimentation such that promising objective values are attained for all objectives. There is no further meaning of the weighting due to the heterogeneity of the objectives involved.

Objective (1) gives the total score of the individual student schedules. Objective (2) yields the total penalty for empty time slots of specific lengths between two consecutive appointments. Objective (3) provides the total penalty for underutilized days. Constraint (4) assigns exactly one of the available room-time slots for tutorials to a student for every course with a tutorial. Constraint (5) defines the appointment indicator, i.e., the occurrence of a lecture or tutorial appointment for a specific student on a specific time slot. Constraint (6) delimits the number of participants in each tutorial appointment to the minimum of the available room capacity and the maximum number of allowed participants. Constraint (7) defines the class count, i.e., the number of lectures and tutorials of a student on a specific day. Constraint (8) provides a lower bound on the class count. Together with objective (3) the lower bound depends on whether one or none class is scheduled for a student on a specific day in which case the lower bound would be diminished. Constraint (9) forces the indicating variable for the series of empty slots to 1 whenever a series of empty slots occurs. Together with objective (3) the variable is set to 0 when such a series of empty slots does not occur.

The three-step procedure for sequentially determining lecture schedules, tutorial schedules, and individual student schedules is subsequently used as part of the GA outline. Hence, the models are utilized as part of a matheuristic.

### GA for university schedule optimization

To cope with the heterogeneity in the objectives resulting from the heterogeneity of stakeholders involved in multi-level university timetabling, we develop a GA solution outline which builds upon the IP formulations derived in Sect. 3.2 and hence represents a matheuristic. Further, we enhance the GA with a metamodel-based search phase. However, for a better understanding, the integration of the metamodel into the GA will be explained afterward in Sect. 3.4.

#### Chromosome design

While a solution to multi-level university timetabling ultimately consists of a (large) set of schedules on every planning level (lectures, tutorials, individual schedules), representing it in such detail would render it useless in terms of processing it in a GA. Hence, due to the computationally intractable representation of a solution of to model $\textbf{SSP}$, we employ a chromosome representation which addresses the foundational solution of model $\textbf{LPP}$ as it lays the ground for both the results attainable from $\textbf{TPP}$ and $\textbf{SSP}$. We choose an encoding scheme based on the LCs $n_{\mathcal S\mathcal D}:=(n_{sd})_{s \in \mathcal S, d \in \mathcal D}$ holding the number of lectures of study program $s \in \mathcal S$ on day $d \in \mathcal D$. Hence, we employ value encoding with $n_{sd} \in \mathbb N_0$ for $s \in \mathcal S, d \in \mathcal D$. Together with the attractive computational times for solving the models $\textbf{LPP}$ and $\textbf{TPP}$, this coarse level of detail of this chromosome representation complies with the practical goal of obtaining a diverse selection of university timetables. This is due to the fact that timetables are developed not out of an existing detailed solution, but rather out of a coarse timetable frame only specified by $n_{\mathcal S\mathcal D}$ without prescribing course assignments prematurely. For a given LC, the concrete lecture, tutorial, and student schedules can then be constructed by solving the corresponding IP formulations from Sect. 3.2.

#### Fitness evaluation

Recalling the seven objective functions (lecturer score $\psi ^{l}_{val}$, ideal lecture number $\psi ^{l}_{num}$, gap-freeness $\psi ^{l}_{gap}$, average student score $\psi ^{t}_{val}$, student score $\psi ^{s}_{val}$, gap-freeness $\psi ^{s}_{gap}$, idle-freeness $\psi ^{l}_{idle}$), we recognize that we can get rid of $\psi ^{l}_{gap}$ (because we have $\psi ^{s}_{gap}$) and of $\psi ^{t}_{val}$ (because we have $\psi ^{s}_{val}$). Hence, $\Psi = (\psi ^{l}_{val}, \psi ^{l}_{num}, \psi ^{s}_{val}, \psi ^{s}_{gap}, \psi ^{l}_{idle})$ must be organized by a fitness consideration for evaluating the quality of the associated LC $n_{\mathcal S\mathcal D}$. We proceed according to the non-dominated sorting algorithm NSGA-II (Deb et al., 2002). In particular, we do not compute an individual fitness value for each individual, but we compare individuals with each other. Comparison is based on the concepts of the nondomination rank (*NDR*) and the crowding distance (*CD*). The *NDR* tells us the level of nondominatedness of the objective vector $\Psi$. It is defined recursively: We have $NDR(\Psi ) = 0$ if there is no other objective vector $\Psi ' \ne \Psi$ which dominates $\Psi$; we have $NDR(\Psi ) = 1$ if there is no other objective vector $\Psi ' \ne \Psi$ which dominates $\Psi$ apart from those with $NDR(\Psi ') = 0$; we have $NDR(\Psi ) = 2$ if there is no other objective vector $\Psi ' \ne \Psi$ which dominates $\Psi$ apart from those with $NDR(\Psi ') \in \{0, 1\}$. To distinguish between two objective vectors $\Psi , \Psi '$ with $NDR(\Psi ) = NDR(\Psi ')$, the crowding distances $CD(\Psi )$ and $CD(\Psi ')$ serve as a diversification-fostering tie-breaker telling us how crowded the regions of $\Psi$ and $\Psi '$ are, respectively. A smaller crowding distance means that the region is more crowded as neighboring individuals are closer. For the purpose of ensuring diversification, hence, a larger crowding distance is preferred. $CD(\Psi )$ is computed as the sum of the objective-specific *CD*s. The objective-specific *CD* is $\infty$ if $\Psi$ exhibits the smallest or largest value for the considered objective; otherwise, the objective-specific *CD* amounts to the absolute distance between the two adjacent objective values divided by the absolute difference between smallest and largest value of the considered objective (for the purpose of normalization). The pseudo-code can be found in the paper introducing NSGA-II (Deb et al., 2002).

#### Initial population

We find the first LC by solving problem $\textbf{LPP}$ from Sect. 3.2.1 with $n_{\mathcal S\mathcal D}$ as decision variables. To obtain a large pool of LCs exhibiting a high degree of diversity we then impose a random drawing of the elements of $n_{\mathcal S\mathcal D}$ for a sufficient number of times. Since there are six time slots per day and no day shall have no lecture, every element $n_{sd}$ for $s \in \mathcal S, d \in \mathcal D$ is a random integer in $\{1, \ldots , 6\}$ such that $\sum _{d \in \mathcal D}n_{sd}$ equals to total number of lectures in the curriculum of study program $s \in \mathcal S$. The initial population is then sent out as the initial mating pool out of which a parent generation is transferred to an offspring generation. Observe that each LC $n_{\mathcal S\mathcal D}$ is turned into a VLC by determining the objective values which result from successively solving the models $\textbf{LPP}(\varvec{n_{\mathcal{S}\mathcal{D}}})$, $\textbf{TPP}(\varvec{occ_{\mathcal{S}\mathcal{I}}})$, and $\textbf{SSP}(\varvec{val_{\mathcal{P}\mathcal{I}}, occ_{\mathcal{P}\mathcal{I}}})$ and associating them with $n_{\mathcal S\mathcal D}$ in a pair $(n_{\mathcal{S}\mathcal{D}}, \psi )$.

#### Genetic operators

Using the current population as a mating pool, solution selection is carried out to determine out of which LCs an offspring chromosome can be generated through recombination and mutation. We generate a fixed number of both recombined and mutated individuals. The new population then corresponds to the offspring generated from the parent generation.

**Fig. 9:**
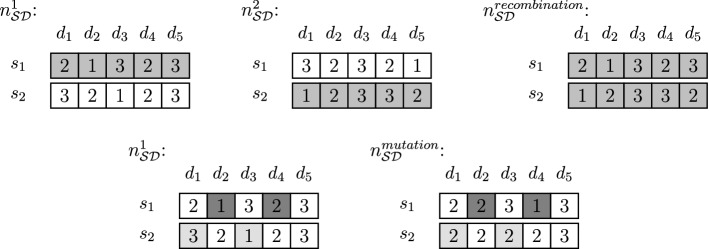
Illustration of recombination and mutation operators

- **selection** Due to the large number of performance measures and different stakeholders, there is a lack of a natural notion of optimality. Hence, survival is possible for all kinds of VLCs and there is low selection pressure within the practical context. Nonetheless, we explicitly take into account all objectives simultaneously as suggested by the NSGA-II algorithm (Deb et al., 2002) and explained in Sect. 3.3.2. To this end, we first induce a sorting of all individuals of the current population using the *NDR*. To distinguish between individuals with equal *NDR*, we utilize the *CD*. Individuals admissible for recombination and mutation are then determined as winners of a binary tournament selection: Individuals with lower *NDR* win against individuals with higher *NDR*; in case of a tie, the individual with larger *CD* is selected. Clearly, various alternatives to this selection strategy are available such as rank selection, or canonical selection.
- **recombination** Two parents are recombined into a single offspring by randomly choosing two individuals $n_{\mathcal S\mathcal D}^1, n_{\mathcal S\mathcal D}^2$ from the mating pool and randomly choosing for each study program $s \in \mathcal S$ whether the corresponding LC components for all $d \in \mathcal D$ come from $n_{\mathcal S\mathcal D}^1$ or from $n_{\mathcal S\mathcal D}^2$. This corresponds to an inter-LC move as shown in the upper part of Fig. 9.
- **mutation** An individual from the parent generation is mutated by randomly selecting over all study programs a fixed number of pairing days to be slightly changed in their number of lectures taking place on these days. For each pairing then the number of lectures on the first day is increased by one, whereas it is decreased by one for the second day. The resulting mutated individual is considered for further consideration only if the number of lectures per day is not zero. This corresponds to an intra-LC move as shown in the lower part of Fig. 9.

The GA is summarized in Algorithm 2. The matheuristic foundation of the algorithm is embodied through subroutine determineSchedules  in Algorithm 3 for determining lecture, tutorial and student schedules using the IP models from Sect. 3.2. The use of the metamodel subroutine employMetamodel  from Algorithm 4 is explained next. We finally remark that for the determination of the (non-) dominance property of an LC $n_{\mathcal S\mathcal D}$ in line 19 of Algorithm 2, we consider $\Psi = (\psi ^{l}_{val}, \psi ^{s}_{val}, \psi ^{l}_{num}, \psi ^{s}_{gap}, \psi ^{s}_{idle})$ as the vector of relevant performance measures. This is due to $\psi ^t_{val}$ and $\psi ^l_{gap}$ both representing only interim performance measures as already explained in Sect. 3.3.2.
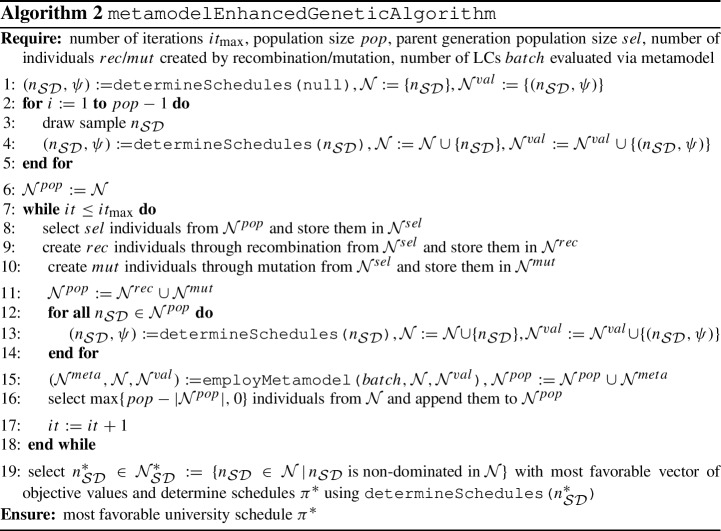

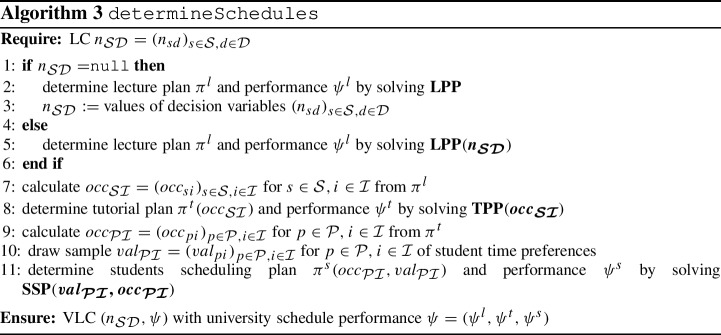

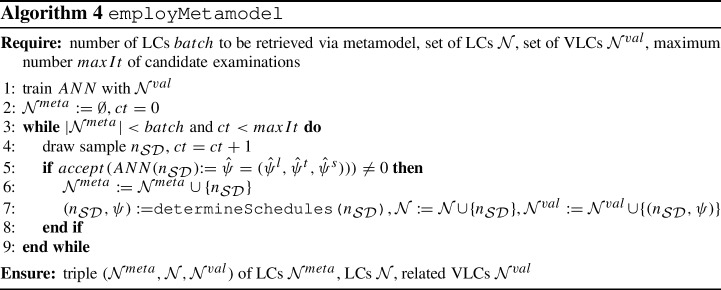


### ANN for estimating university schedule performance

**Fig. 10:**
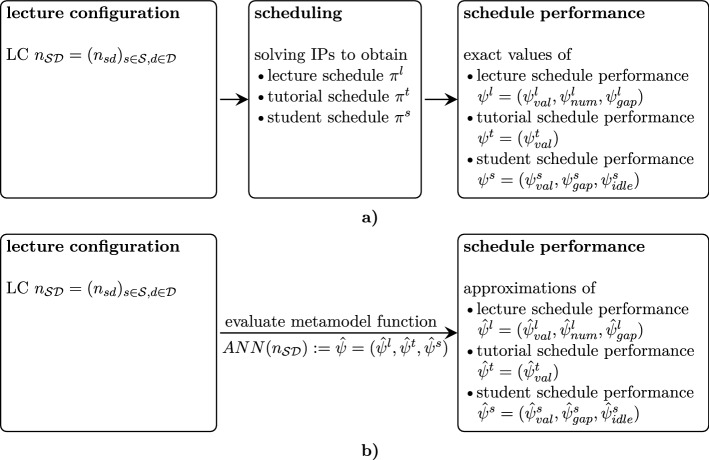
University timetabling workflow **a** without and **b** with metamodel use for determining the estimated quality of a lecture configuration schedule

The subroutine employMetamodel from Algorithm 4 enhances the GA in Algorithm 2 by the possibility to pre-examine a large number of additional LCs in an approximate fashion so as to remove those LCs exhibiting poor approximate performance. Hence, we employ the ANN as a metamodel for the purpose of functional regression in order to eliminate LCs from further consideration when they are expected to yield poor performance as suggested by the ANN metamodel at an early stage. Figure 10 illustrates the role of such a metamodel in terms of bypassing the necessity of directly solving the IP formulations to obtain exact values of the performance measures. To this end, we utilize the ANN as a metamodel for estimating the performance measures resulting from a randomly drawn LC $n_{\mathcal{S}\mathcal{D}}$. For a large number of such LCs, approximate schedule performance is obtained by evaluating the metamodel function. When inferior approximate schedule quality is identified for an LC from the computationally cheap ANN function evaluation, then this LC is excluded from further consideration. Otherwise the LC is turned into a VLC by running the computationally expensive subroutine determineSchedules ($n_{\mathcal{S}\mathcal{D}}$).

We next explain for a LC how to decide upon its acceptance for further consideration or its rejection due to estimated inferiority To this end, recall that $\psi ^{l}_{val}$ and $\psi ^{s}_{val}$ are to be maximized, whereas $\psi ^{l}_{num}$, $\psi ^{s}_{gap}$, and $\psi ^{l}_{idle}$ are to be minimized. Moreover, let $val^l$, $val^s$, $num^l$, $gap^s$, and $idle^l$ be the individually best objective values encountered so far.

We first define the number of objective quality violations due to exceeding a 50 % safety margin as

$$\begin{aligned} n_{viol}(\hat{\Psi }) =&\textbf{1}_{[-\infty , 0.5val^l]}(\hat{\psi }^{l}_{val}) + \textbf{1}_{[-\infty , 0.5val^s]}(\hat{\psi }^{s}_{val})+\\&\textbf{1}_{[1.5num^l, \infty ]}(\hat{\psi }^{l}_{num}) + \textbf{1}_{[1.5gap^s, \infty ]}(\hat{\psi }^{s}_{gap}) + \textbf{1}_{[1.5idle^s, \infty ]}(\hat{\psi }^{s}_{idle}). \end{aligned}$$

Then the indicator for acceptance is defined as follows:

F1 $$\begin{aligned} {accept}(\hat{\Psi }) := {\left\{ \begin{array}{ll} 1 &{} n_{viol}(\hat{\Psi }) \le viol_{max}\\ 0 &{} \text {else} \end{array}\right. } \end{aligned}$$

Hence, after valuation with the estimated objective function outcomes, a LC is only further considered when all but at most $viol_{max}$ objective estimates are within a 50 % margin relative to the best found individual objectives so far. Since we have five objectives, $viol_{max} \in \{2, 3\}$ is reasonable. Observe that the relatively large safety margin of 50 % for each objective in the fitness function definition also intends to limit the risk for falsely excluding LCs which would be worthwhile for future consideration.

**Fig. 11:**
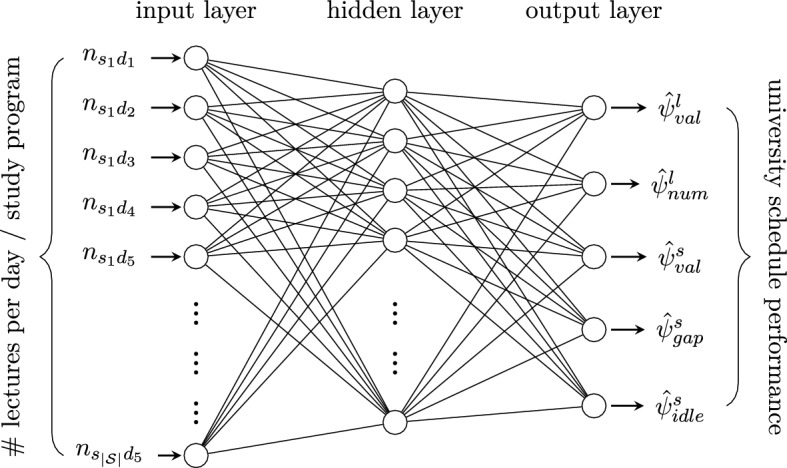
ANN as a metamodel for functional regression in university timetabling

We next describe the elements comprising the ANN as used in the GA for university timetabling. The network function of the ANN is updated through updating its weights with every call of employMetamodel. The ANN structure is schematically depicted in Fig. 11.

- **inputs (independent variables)** components of LC $n_{\mathcal{S}\mathcal{D}}=(n_{sd})_{s \in \mathcal S, d \in \mathcal D}$, i.e., the number of input neurons is $|\mathcal S| \cdot |\mathcal D| = 5 \cdot |\mathcal S|$
- **outputs (target variables)** predictions $\hat{\Psi }= (\hat{\psi }^{l}_{val}, \hat{\psi }^{s}_{val}, \hat{\psi }^{l}_{num}, \hat{\psi }^{s}_{gap}, \hat{\psi }^{s}_{idle})$ for objective values used in the fitness function evaluation, i.e., the number of output neurons is 5.
- **data basis** VLCs stored in $\mathcal N^{val}$

We conclude that the developed solution procedure is a matheuristic building upon an overarching GA which represents solutions in the form of LCs. It repeatedly solves IP models to obtain lecture schedules, tutorial schedules, and individual student schedules, and to transform the LCs into VLCs. The GA is augmented by the possibility to repetitively train and utilize an ANN as a metamodel for estimating schedule performance at low computational costs extending the range of LCs to be investigated. The method outputs a set of non-dominated LCs which can be further processed by the administrative personnel for selecting the most suitable LC according to the institution’s policy on assessing schedule quality.

## Computational experiments

We conduct a series of experiments to evaluate the developed outline in terms of tracing the contributions of its constituent elements towards providing university schedules on all three planning levels (lectures, tutorials, individual student schedules). To ensure that the method can be utilized in practice by administrative timetabling personnel, we do not impose advanced computing requirements: Computational experiments are performed on a personal computer with Intel Core 3.2 GHz processor and 16 GB RAM under Microsoft Windows 10 (64-bit). Algorithms are coded in Python 3.8; IP models are coded in Python using the docplex modeling library and solved using the IBM ILOG CPLEX 20.1.0 solver; ANNs are implemented as multi-layer perceptron regressors using Python’s scikit-learn library for machine learning.

### Instances

Computational tests are performed over 21 instances whereof one instance is a real-world instance and the remaining 20 instances are generated artificially to examine the applicability and limitations for different instance sizes.

#### Real-world instance

Originating from the practical task of timetabling arising at the Department of Economics and Management at the Karlsruhe Institute of Technology in Germany, we consider the following real-world instance as a base case:

- 4 study programs with 452, 122, 413, 102 students, respectively, i.e., 1089 students
- study program curricula are composed of 22 courses requiring 41 lecture classes
- 9 courses offer tutorials requiring 170 tutorial classes
- 30 time slots are available with 6 time slots on each of the 5 week days
- 106 room-time slots for lectures are available with capacities between 105 and 734 seats; only 30 room-time slots can accommodate more than 450 students, i.e., rooms with enough capacity represent a bottleneck resource
- 279 room-time slots for tutorials are available with capacities between 24 and 30 seats; the upper bound of 30 is a requirement on the maximum tutorial class size
- lecturers and students provide preferences for each time slot as a score from $\{1, 2, 3, 4, 5\}$; historical records on student preferences for each time slot are used for tutorial planning

#### Generated instances

To solidify the computational analysis, we develop an instance generator producing randomly drawn data instances upon providing the generator with the number $|\mathcal S| \in \{2, 4, 6, 8\}$ of study programs and the average number $avg stud_s \in \{50, 100, 200, 400, 800\}$ of students in each study program as constituting instance data components. These quantities allow us to generate $4 \cdot 5 = 20$ diverse and realistic instances of varying size under the following assumptions ensuring schedule feasibility on all planning levels:

- student scores for time slots follow a normal distribution with mean 2.5 and standard deviation 0.25; first and last time slots of a day exhibit a mean of 2.0
- the number of students in a study program is drawn from a normal distribution with mean $avg stud_s$ and standard deviation $0.25 \cdot avg stud_s$ and rounded to the next integer
- the number of lecture rooms is $2\cdot |\mathcal S|$; the number of time slots per room is randomly drawn from $\{10, 11, \ldots , 15\}$; the capacity of each room is drawn as a uniformly distributed value in the range of the minimum of the number of students in a study program and the sum of the two largest number of students in a study program
- the number of tutorial rooms is $8\cdot |\mathcal S|\cdot \frac{avg stud_s}{50}$; the number of time slots per room is randomly drawn from $\{12, 13, \ldots , 18\}$; the capacity of each room is 30
- the number of courses is $\lceil 4.5 \cdot |\mathcal S|\rceil$; the number of online courses is randomly drawn from $\{1, 2, \ldots , |\mathcal S|\}$; all courses have 2 lectures per week except for $\lceil \frac{|\mathcal S|}{2}\rceil$ courses having 1 lecture per week and $\lceil \frac{|\mathcal S|}{2}\rceil$ courses having 3 lectures per week
- each study program receives six randomly drawn courses; re-distributions are executed in case that a course initially receives no study program; in the latter case study programs are shifted to empty courses from courses with more than two study programs
- each study program offers tutorials in 3 courses; the number of tutorials offered for each course *c* is $15 \cdot |\mathcal S_c| \cdot \frac{avg stud_s}{50}$

### Algorithmic settings

Following the multi-objective selection strategy prescribed by NSGA-II, we employ the GA in Algorithm 2 from Sect. 3.3 with $it_{max}=10$ iterations, initial population size $pop=25$, parent generation population size $sel=10$, $rec=5$ and $mut=5$ individuals created by recombination and mutation, respectively, and $batch=10$ LCs evaluated via ANN metamodel. For ANN-related probing of LCs, we use $viol_{max} = 2$ in the acceptance indicator function $accept(\hat{\Psi })$ (cf. Equation (F1) in Sect. 3.3.2) as the maximum number of objectives allowed to lie outside the 50 % performance guarantee. For solving the IP models $\textbf{LPP} / \textbf{LPP}(\varvec{n_{\mathcal{S}\mathcal{D}}})$, $\textbf{TPP}(\varvec{occ_{\mathcal{S}\mathcal{I}}})$, and $\textbf{SSP}(\varvec{val_{\mathcal{P}\mathcal{I}}, occ_{\mathcal{P}\mathcal{I}}})$, we employ the default relative MIP gap tolerance of the IBM ILOG CPLEX 20.1.0 solver amounting to 0.0001.

### Results

We subsequently present the computational results by addressing the following topics of evaluation: schedule quality, number of non-dominated LCs and their accrual over the course of the algorithm, and computational times. Results are discussed jointly for the real world instance and the randomly generated instances, i.e., there is no need to differentiate the findings based on the instances’ origins. Hence, we find that the hypothetical instances exhibit realistic behavior and contribute to a comprehensive view required for the overall analysis of the methodology’s performance.

#### Schedule quality

**Fig. 12:**
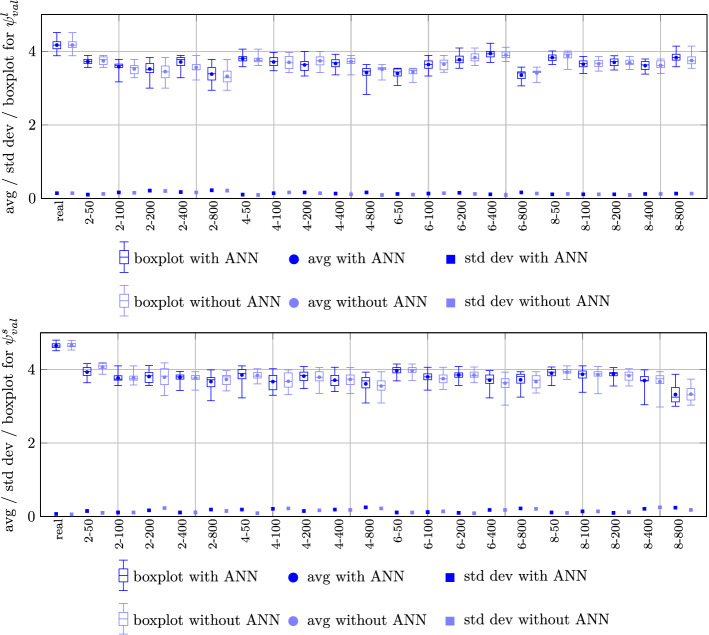
Statistics for lecturer score (top), student score (bottom) for non-dominated LCs

We first evaluate the schedule quality related to the schedules of non-dominated LCs with respect to both score-related and curriculum-related statistics. Figure 12 illustrates the score-related statistics for lecturers (top) and students (bottom), respectively. With a maximum score of 5, both lecturer and student scores are highly satisfying. In particular, we see that in the real world instance score values consistently larger than 4 are obtained, whereas in the artificial instances the value is between 3 and 4. This points to the fact that in practice both lecturers and students exhibit flexibility in terms of a rather generous rating of time slots. We do not observe a substantial difference or tendency in terms of an additional benefit of enhancing the GA through ANN metamodels. However, as will be seen in Sect. 4.3.2, the number of non-dominated LCs is increased significantly through employing ANN metamodels.

**Fig. 13:**
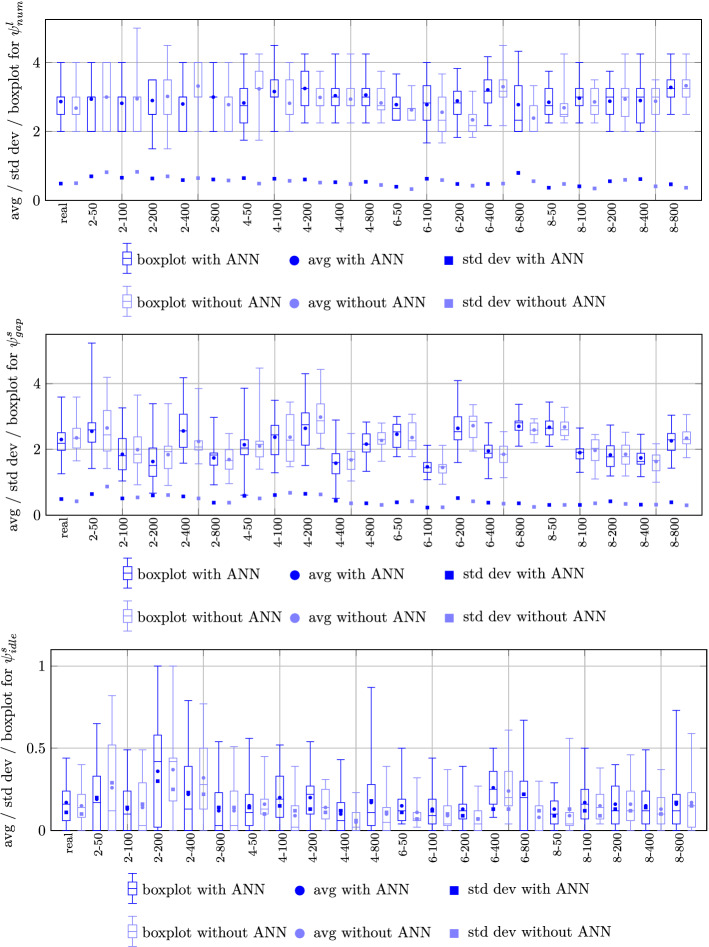
Statistics for deviation from ideal number of lectures per study program (top), number of empty time slots per student (middle), number of days with at most one class per student (bottom) for non-dominated LCs

Figure 13 illustrates the curriculum-related statistics for the deviation from the ideal number of lectures (top), empty time slots (bottom), and idle days (bottom), respectively. Observe that these values are normalized to a per study program and per student perspective, respectively. Concerning the number of deviations from the ideal number of 2 lectures per day, we remark that for a study program with an uneven number of lectures per week an unavoidable contribution of 1 to the objective value occurs. Hence, the results seen at the top of the figure are consistently very satisfying in terms of scheduling the desirable amount of lectures on nearly every day. In case of high number of students per study program, the number of deviations from the ideal number rises slightly due to limited availability of large lecture halls of sufficient capacity. Also the number of empty slots hovering around the value of 2 is very tolerable in terms of schedule quality as perceived individually. Finally, the number of days with at most one class is consistently below 0.5 per student. Thus, workload is balanced homogeneously over the week for the overwhelming majority of students.

From the evaluation of the individual performance measures for obtained schedules on the lecture, tutorial, and individual student level, we conclude that schedule quality related to non-dominated LCs is very high and points towards a large degree of reliability in terms of serving both individual as well as normative requirements on university schedules. We also remark that the threshold requirement criterion of eliminating all VLCs with inferior performance in more than $viol_{max}$ objectives successfully leads to relatively narrow boxplots indicating that non-dominated LCs which may have an unacceptable objective in one of the objectives are safely eliminated from further consideration.

#### Number of non-dominated LCs and their accrual over the course of the algorithm

**Fig. 14:**
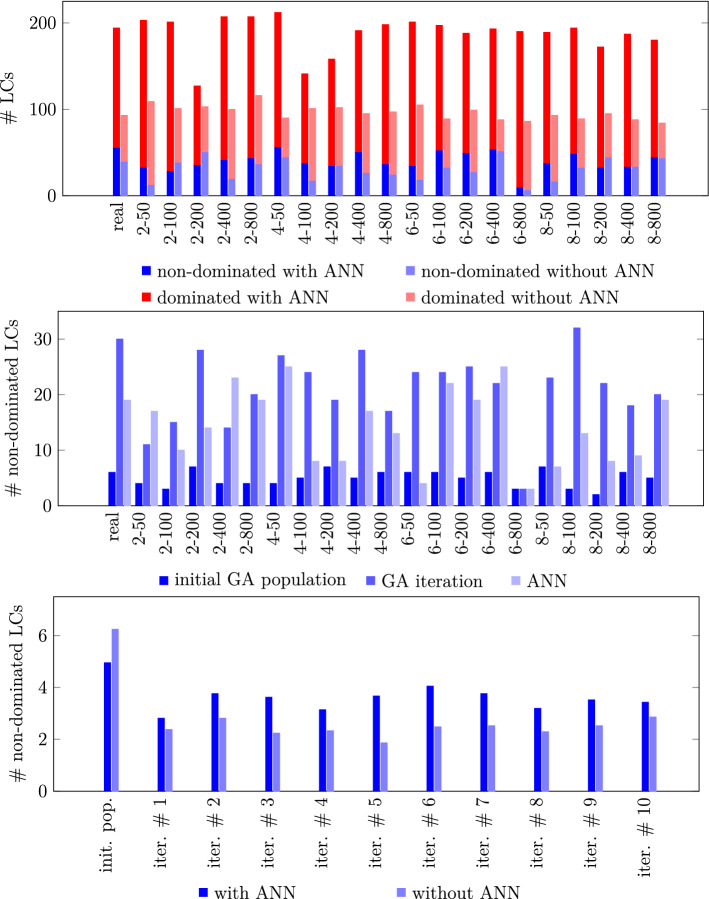
Number of non-dominated and dominated LCs (top), accrual of non-dominated LCs over algorithm parts (middle), accrual of non-dominated LCs over iterations (bottom)

As shown by the blue bars in comparison to the red bars in the top of Fig. 14, both with and without the use of an ANN metamodel, the number of obtained non-dominated LCs is sufficiently high to allow administrative personnel to find a suitable set of lecture and tutorial schedules which will serve as the basis for the student schedules at semester start. With an average of 27 % of non-dominated LCs, on average the ratio between dominated and non-dominated LCs is approximately 3:1. In addition to the non-dominated schedules generated over the GA iterations, the utilization of the ANN metamodel generates a significant number of additional non-dominated LCs which may serve as candidates for the university schedule as displayed in the middle of Fig. 14. A third place of origin lies in the initial population generation of the GA albeit to a much smaller extent. Finally, from the bottom of Fig. 14, we see that non-dominated LCs are found consistently over all iterations. When no ANN metamodel is employed, the share of non-dominated LCs emanating from the initial population is larger than in case of the ANN metamodel utilization. Therefore, it is an advantage of the ANN metamodel approach to produce non-dominated LCs at a rather constant rate over the iterations depending on the number *batch* of promising LCs to be retrieved. As a consequence, once data from the GA is available, an ANN can be trained and subsequently used as the basis for generating LCs. The advantage of ANNs is that their (computationally cheap) evaluation gives a first hint towards an LC’s potential with respect to prospectively creating high quality schedules. Overall, once enough data on the relation between LCs and their respective valuations is learned by the ANN, this grants the option for a significant reduction of computational times required for suggesting promising LC candidates as compared to the rather random proposals suggested via GA recombination.

#### Computational times

**Fig. 15:**
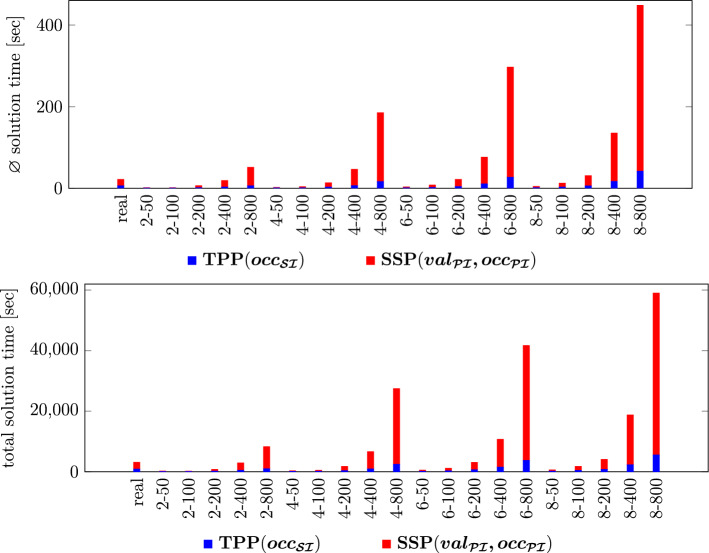
Average solution times per IP model instance (top) and over all IP model instances (bottom) in seconds required by CPLEX solver

Solving model $\textbf{LPP} / \textbf{LPP}(\varvec{n_{\mathcal{S}\mathcal{D}}})$ takes less than two seconds even for the most difficult case of 8 study programs with 800 students per study program. Therefore, we restrict our attention to the computational times required for solving $\textbf{TPP}(\varvec{occ_{\mathcal{S}\mathcal{I}}})$ and $\textbf{SSP}(\varvec{val_{\mathcal{P}\mathcal{I}}, occ_{\mathcal{P}\mathcal{I}}})$, respectively. We recall that models are solved several hundreds of times during GA execution and during LC valuation when an LC has been identified as promising by the ANN metamodel. The top of Fig. 15 demonstrates that solution times required for solving a single IP model instance are fully acceptable for up to 200 students enrolled per study program irrespective of the number of study programs. However, results for 400 and especially 800 students in a study program then clearly demonstrate the computational limitations of the matheuristic outline. While every instance of $\textbf{TPP}(\varvec{occ_{\mathcal{S}\mathcal{I}}})$ can easily be solved in several seconds and in all cases in less than 45 s on average, the time required to solve $\textbf{SSP}(\varvec{val_{\mathcal{P}\mathcal{I}}, occ_{\mathcal{P}\mathcal{I}}})$ climbs dramatically for too large number of students. The limitation of the matheuristic for large problem sizes follows from the bottleneck component of having to solve $\textbf{SSP}(\varvec{val_{\mathcal{P}\mathcal{I}}, occ_{\mathcal{P}\mathcal{I}}})$ to obtain hundreds of individual student schedules. This is confirmed by the related cumulated runtime at the bottom of Fig. 15. For 8 study programs and 800 students, i.e., the largest setting, computational time amounts to approximately 17 hours when averaged over the runs with and without ANN support, respectively, due to nearly 400 s on average required for solving one instance of $\textbf{SSP}(\varvec{val_{\mathcal{P}\mathcal{I}}, occ_{\mathcal{P}\mathcal{I}}})$. While this can be considered acceptable for a problem to be solved once in every semester, it also suggests that computational times for more than 800 students will be prohibitive. We conclude that for student numbers in these regions, (tailored) heuristic or metaheuristic approaches are needed in order to obtain solutions especially for problem $\textbf{SSP}(\varvec{val_{\mathcal{P}\mathcal{I}}, occ_{\mathcal{P}\mathcal{I}}})$ more efficiently.

## Key findings for best practices

The process of developing the metamodel-enhanced multi-level university timetabling approach in Sect. 3 and applying it to a range of settings in the computational experiments in Sect. 4 has yielded several insights of both technical and practical nature. We gather these learnings to promote the establishment of best practices for multi-level university timetabling. The findings may help both university timetabling personnel when confronted with specific issues arising at the juncture between lecture, tutorial, and student schedule planning as well as academic researchers when seeking for advice on existing research directions and information on methodological approaches along with their benefits, burdens, and performances.

Nomenclature and terminology are crucial to all communications between stakeholders and represent a fundamental precondition for devising models and solution methods for university timetabling. Likewise, high schedule quality can only be reached when schedule performance criteria and mandatory normative didactic schedule properties are precisely specified.

The structured approach of solving problems at different planning levels allows to accumulate improvements in schedule quality consistently over these levels. The multi-level approach also yields insight into more detailed causes and effects relations as they can be traced to separate planning stages. Nonetheless, each one of the models $\textbf{LPP}$, $\textbf{TPP}$, $\textbf{SSP}$ can also be used as a standalone model serving a specific purpose, e.g., $\textbf{LPP}$ establishes skeleton schedules whereas $\textbf{SSP}$ provides customized individual schedules. From an optimization perspective, the schedules obtained by the matheuristic realize the potential intrinsic to the combinatorial nature prevalent at each level. Clearly defining the interfaces between the models then paves the way for an overall architecture of university scheduling. Such an architecture bears the advantage that it can be tailored towards needs and requirements of the institution’s scheduling process. Hence, it fosters a profound comprehension of the overall task and the interconnectedness of subtasks within a seamless outline.

The main function of the GA lies in the generation of a sufficient number of non-dominated schedule candidates to be handed over to the scheduling personnel’s discretion. Hence, the GA-based determination of schedules within a pool of solution candidates allows to maintain a selection of diverse schedules with respect to the different performance measures resulting from the heterogeneity of stakeholders. In particular, this results in a GA implementation exhibiting low selection pressure favoring a balance between objectives. Utilizing the ANN is strongly supportive to the function of gathering non-dominated schedules as it steadily produces them over the course of the GA iterations.

As seen from the development process of the models, keeping them open and adaptable to changes in circumstances (e.g., different classroom formats) or requirements (e.g., additional performance criteria) is likely to be beneficial in the future. As learned during model evolution, a systematic way to incorporate additional constraints lies in translating them into logical expressions and transforming these into disjunctive normal form. Careful attention should be given to the modeling of real world bottleneck resources. In our institution, this concerned the scarcity of large lecture rooms; in other cases it may be another factor (e.g., lecturer availability) which must be specifically addressed in modeling.

$\mathcal{N}\mathcal{P}$-hardness becomes an issue for model $\textbf{SSP}$ in case of more than 500 students. Research on relieving this computational burden is needed: Developing and maintaining ANN metamodels represents a promising direction, but requires a more systematic analysis and maintenance of a larger data basis to be used reliably. Nonetheless, even if predicting performance criteria received upon solving $\textbf{SSP}$ could be approximated by an ANN with high precision, $\textbf{SSP}$ still must be solved for the final student schedules. Hence, also tailored (meta-) heuristic methods are required. Overall, employing model $\textbf{SSP}$ leads to high acceptance of the overall approach, since students actively participate in the schedule generation process by submitting their preferences for tutorial appointments.

Results obtained for the real world instance are consistently competitive with results from hypothetical instances. From the convincing quality of the objective values, we conclude that in comparison to manually planned schedules, there is wide room for improvement through customization of individual schedules. Clearly, these improvements are assembled from improvements on all levels of the planning process and ultimately evoked by the use combinatorial optimization methods.

## Conclusion and outlook

With the goal of providing a practically viable methodology for university timetabling, we have devised a matheuristic allowing for a multi-criteria consideration in the multi-level generation of schedules for lectures, tutorials, and individual students. The matheuristic is enhanced by an ANN metamodel such that exact analysis efforts—as encountered when solving IP models—can be reduced while still facilitating the analysis of wide portions of the LC search space. The developed methodology is capable of keeping up with contemporary needs of academic institutions such as offering digital courses or using large lecture halls efficiently as well as granting individual high-quality schedules to students on a customized basis. The outline is designed hierarchically and segregates the planning process into lecture planning, schedule planning, and individual student schedule planning. The division of planning onto several levels yields the opportunity to include different schedule performance measures relevant to the different stakeholders involved in university timetabling, namely students, lecturers, and administrative personnel responsible for didactic adequacy of established schedules. Hence, we comprehensively account for both score-related and curriculum-related objectives in multi-level multi-criteria university scheduling. Moreover, tackling a multi-criteria setting through a GA which carries out the selection of individuals according to different objectives represents a way of ensuring diversification in terms of viewing schedule performance from several perspectives. Nonetheless, the complexity of the problem setting requires a hierarchical outline which leaves several degrees of freedom to the decision maker, i.e., the final adoption of the university schedule to be realized remains in the hands of the decision maker’s assessment of the resulting vector of objective values.

Several avenues for future research are identified: First, like for all GA implementations, the method’s sensitivity to prescribed definitions (e.g., numerical parameters, fitness function, or genetic operators) is of interest when this would lead to qualitatively different solutions. Therefore, further experimentation is required. Second, enhancing the approximation quality of the ANN metamodel over time with restricted effort for incorporating training and testing data represents another issue worthwhile of investigation. This could be achieved through integrating the method into the practical planning environment such that the data basis automatically grows from semester to semester. Third, the dependency on solution times for the IP models currently represents a computational bottleneck and hence may serve a starting point for extending the proposed methodology by metaheuristic solution methods for the IP model $\textbf{SSP}(\varvec{val_{\mathcal{P}\mathcal{I}}, occ_{\mathcal{P}\mathcal{I}}})$. This would be needed to facilitate a more efficient scan of broader search regions. Finally, with respect to practical applicability, a resolution must be made on how to cope with multiple performance criteria. In this respect, being able to process multi-criteria information in a practically meaningful way poses another challenge for which different solution concepts from multi-criteria optimization must be compared to each other in the setting of university scheduling. In particular, resolving the trade-offs between several schedule criteria certainly poses a challenge which must be addressed in very close collaboration with experienced university timetabling personnel.
